# Targeted metabolomics reveals proline as a major osmolyte in the chemolithoautotroph *Sulfurimonas denitrificans*

**DOI:** 10.1002/mbo3.586

**Published:** 2018-02-09

**Authors:** Florian Götz, Krista Longnecker, Melissa C. Kido Soule, Kevin W. Becker, Jesse McNichol, Elizabeth B. Kujawinski, Stefan M. Sievert

**Affiliations:** ^1^ Biology Department Woods Hole Oceanographic Institution Woods Hole MA USA; ^2^ Department of Marine Chemistry and Geochemistry Woods Hole Oceanographic Institution Woods Hole MA USA; ^3^ Present address: Department of Biological Sciences University of Southern California Los Angeles CA USA

**Keywords:** environmental stress, metabolism, metabolomics, microbial ecology, osmoregulation, sulfurimonas

## Abstract

Chemoautotrophic bacteria belonging to the genus *Sulfurimonas* in the class *Campylobacteria* are widespread in many marine environments characterized by redox interfaces, yet little is known about their physiological adaptations to different environmental conditions. Here, we used liquid chromatography coupled with tandem mass spectrometry (LC‐MS/MS) in a targeted metabolomics approach to study the adaptations of *Sulfurimonas denitrificans* to varying salt concentrations that are found in its natural habitat of tidal mudflats. Proline was identified as one of the most abundant internal metabolites and its concentration showed a strong positive correlation with ionic strength, suggesting that it acts as an important osmolyte in *S. denitrificans*. 2,3‐dihydroxypropane‐1‐sulfonate was also positively correlated with ionic strength, indicating it might play a previously unrecognized role in osmoregulation. Furthermore, the detection of metabolites from the reductive tricarboxylic acid cycle at high internal concentrations reinforces the importance of this pathway for carbon fixation in *Campylobacteria* and as a hub for biosynthesis. As the first report of metabolomic data for an campylobacterial chemolithoautotroph, this study provides data that will be useful to understand the adaptations of *Campylobacteria* to their natural habitat at redox interfaces.

## 1 INTRODUCTION

*Campylobacteria* (formerly classified as *Epsilonproteobacteria*, Waite et al., 2017) are globally abundant in many oxygen‐deficient and sulfide‐rich marine and terrestrial environments where they contribute to chemolithoautotrophic production and the cycling of sulfur and nitrogen (Campbell, Engel, Porter, & Takai, 2006; Labrenz, Jost, & Jürgens, 2007; Sievert & Vetriani, 2012). In particular, the genus *Sulfurimonas* is widespread and representatives have been found in a variety of predominantly marine environments, including hydrothermal vents, sulfidic sediments, the redoxcline of euxinic basins, and oil fields (Han & Perner, 2015). Yet, their adaptations to the chemical conditions in these different environments are not well understood.

*Thiomicrospira denitrificans* (reclassified as *Sulfurimonas denitrificans* (Takai et al., 2006) was originally isolated from tidal mudflats of the Dutch Wadden Sea using a nitrate‐limited chemostat with thiosulfate as the electron donor (Timmer‐Ten Hoor, 1975). These mudflats are characterized by significant variations in salinity, requiring organisms living in this environment to have osmoregulatory strategies to cope with these changes. To adapt to high salt concentrations and maintain osmotic equilibrium with the environment, microorganisms use one of two fundamentally different strategies: either the so‐called salt‐in‐cytoplasm mechanism or the compatible solute mechanism (Sleator & Hill, 2001; Wood et al., 2001). For the salt‐in‐cytoplasm mechanism, potassium chloride at molar concentrations accumulates in the cytoplasm to adjust the osmolarity inside the cell to equal that of the outside environment. This strategy also requires the adaptation of the enzymatic machinery to the high internal salt concentration, and is mainly observed in extremely halophilic bacteria and archaea. In contrast, the compatible solute mechanism uses uncharged, highly water‐soluble organic compounds to cope with osmotic stress—commonly known as osmolytes. The production of osmolytes usually does not require specific adaptations of the intracellular enzymatic machinery, and microorganisms using this strategy are generally adapted to a broader range of salt concentrations. Bacteria, for example, often use the amino acid proline, which can be synthesized from glutamate or ornithine (Fichman et al., 2014), as an osmolyte in response to increased external salt concentrations (Christian, 1955a,b; Fichman et al., 2014; Sleator & Hill, 2001). However, at present the osmoregulatory mechanisms in chemoautotrophic *Campylobacteria* are not known.

Targeted and untargeted metabolomics approaches (cf. Patti, Yanes, & Siuzdak, 2012) have been used to gain information about the metabolome of plants, phytoplankton, and heterotrophic bacteria (Fiore, Longnecker, Kido Soule, & Kujawinski, 2015; Longnecker, Kido Soule, & Kujawinski, 2015; Sawada et al., 2009), while studies of chemolithoautotrophic bacteria are so far limited to acidophilic bacteria used in biomining applications (Martínez et al., 2013). In this study, we have used *Sulfurimonas denitrificans* as a representative model organism to investigate the first metabolome of widely distributed, neutrophilic chemolithoautotrophic *Campylobacteria*, complementing and verifying biochemical pathways inferred from the genome and to assess the adaptations of *S. denitrificans* to osmotic stress.

## 2 MATERIALS AND METHODS

### 2.1 Cultivation of *Sulfurimonas denitrificans*

*Sulfurimonas denitrificans* was grown in a triplicate chemostat setup using an anaerobic saltwater medium based on Timmer‐Ten Hoor (1975). The chemostat was kept anaerobic using a N_2_:CO_2_ (80:20) gas mixture that was continuously supplied. The dilution rate of the chemostat was ~0.88 per day. One experiment started with no changes in the ionic strength compared to the base medium (180 mM, 0 g/L NaCl) and cells were harvested after 96 hr (Figure S1). Subsequently, sodium chloride was added to the reservoir (20 g/L NaCl) to increase the ionic strength in the medium to 520 mM. Cells were harvested again when cell numbers stabilized after ~240 hr (Figure S1). An additional experiment was carried out at an ionic strength of 350 mM (containing 10 g/L NaCl) (Figure S2). All nine samples were prepared for the targeted metabolomics analysis.

### 2.2 Isolation and analysis of metabolites

**Figure 1:**
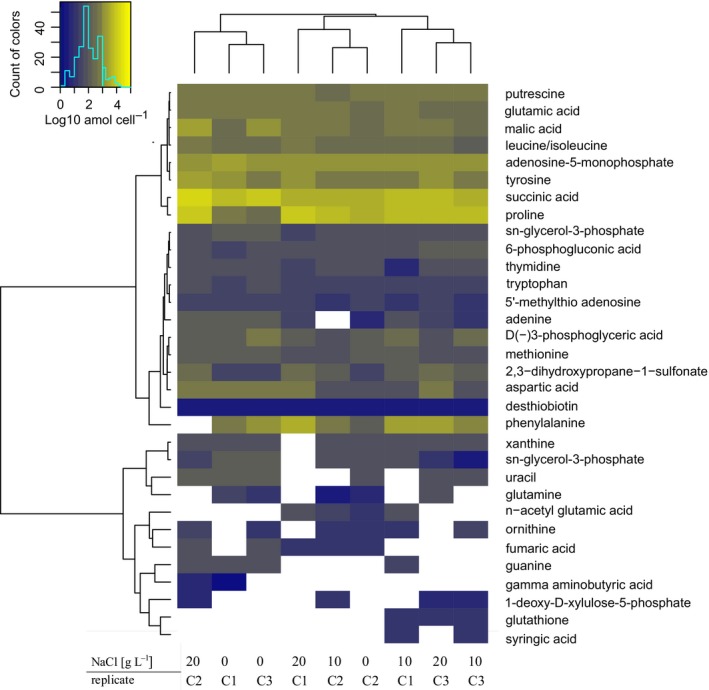
Heatmap showing the most abundant intracellular metabolites at three different sodium chloride concentrations (0, 10, and 20 g/L NaCl). C1, C2, and C3 corresponds to replicate chemostats run at each salt concentration. Concentrations of each metabolite are given as log_10_ attomol per cell. Metabolites only detected in one replicate are not included

**Figure 2:**
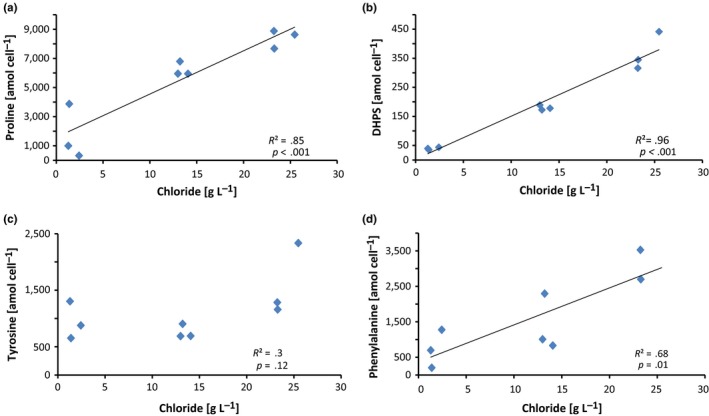
Pearson's correlation between the intracellular metabolite concentration in amol per cell and the measured chloride ion concentration in g/L. Significant positive correlations were found for (a) proline (*R* ^2^ = .85, *p* < .001), and (b) 2,3‐dihydroxypropane‐1‐sulfonate (*R* ^2^ = .96, *p* < .001). While there was a positive trend for (c) tyrosine (*R* ^2^ = .3, *p* = .12), the correlation was not statistically significant. A positive correlation was also found for phenylalanine (*R* ^2^ = .68, *p* = .01) (d). Note: For one of the samples, the concentration of phenylalanine was above the saturation of the detector and thus was not included in the analysis

Metabolites were extracted from approximately 10^10^ cells using methods described in Kido Soule, Longnecker, Johnson, and Kujawinski (2015). Metabolites were identified by running a triple quadrupole mass spectrometer using positive (“pos”) and negative (“neg”) ion‐switching in selected reaction monitoring mode. The heatmap in Figure 1 was calculated using R with Ward clustering and Euclidean distance (Warnes et al., 2014, 2016). Concentrations of each metabolite are given as log_10_ attomol per cell. For Pearson's correlation of metabolites to chloride concentration, samples were 1,000‐fold diluted prior to the measurement of sodium chloride on an ion chromatograph (ICS‐2000, DIONEX) using a DIONEX ASRS 300 4‐mm P/N 064554 column (Figure 2).

### 2.3 Isolation of lipids and lipid analysis

Intact polar lipids (IPLs) were extracted from *S. denitrificans*, grown at different NaCl concentrations (0, 10, 20 g/L), using a modified Bligh and Dyer extraction (Bligh & Dyer, 1959; Popendorf, Fredricks, & Van Mooy, 2013) with 2,4‐dinitrophenyl modified phosphatidylethanolamine diacylglycerol (DNP‐PE‐C_16:0_/C_16:0_‐DAG; Avanti Polar Lipids Inc., Alabaster, AL) as internal standard. IPLs were analyzed by normal phase HPLC‐MS on an Agilent 1200 HPLC (Agilent, Santa Clara, CA, USA) coupled to a Thermo Q Exactive Orbitrap high‐resolution mass spectrometer (ThermoFisher Scientific, Waltham, MA, USA). Chromatography was as described by Popendorf et al. (2013) and mass spectrometry conditions were modified after Collins, Edwards, Fredricks, and Van Mooy (2016). Lipid abundances were corrected for response factors of adequate standards. The abundances of IPLs with phosphatidylglycerol (PG) and PE head groups were corrected by the relative responses of commercial DAG‐C_16:0_/C_16:0_ standards with the respective head group (Avanti Polar Lipids, Inc., Alabaster, AL, USA). Individual PG‐ and PE‐DAG lipids were identified by the total number of acyl carbons and double bonds (C_*n*:*m*_, where *n* indicates the number of carbon atoms and *m* the number of double bonds). The detailed molecular structure of fatty acids cannot be identified by positive ion HPLC‐MS.

## 3 RESULT AND DISCUSSION

### 3.1 Detected metabolites

Using a chemostat we investigated the metabolome of *Sulfurimonas denitrificans* under steady‐state conditions in response to increasing salt concentrations. Cultivation began with an artificial seawater medium with an ionic strength of 180 mM (containing 0 g/L NaCl), and ending with an ionic strength of 520 mM (containing 20 g/L NaCl). Biomass was harvested when cell numbers stabilized (Figures S1 and S2, red arrows), indicating that steady‐state conditions had been reached and that cells had adapted to the respective ionic strength (180, 350, and 520 mM). The targeted metabolomics approach allowed us to connect metabolomics data with previous enzymatic and genomic studies of *S. denitrificans* (Hügler, Wirsen, Fuchs, Taylor, & Sievert, 2005; Sievert et al., 2008) and thus to verify and predict biochemical pathways. The seven most abundant metabolites identified among the 105 tested compounds were putrescine, glutamic acid, leucine/isoleucine, adenosine 5′‐monophosphate, tyrosine, succinic acid, and proline (Figure 1). These compounds were consistently identified in all replicates and at all salt concentrations. Some intracellular metabolites, however, were not always detected among the replicates, likely reflecting their fast turnover inside the cell, as their concentrations were generally low (Figure 1). In addition, one of the three replicates at 180 mM ionic strength (chemostat 2) showed a different profile compared to the other two, which we attribute to biological variation. In general, the intracellular metabolites showed less variation among replicates than the extracellular metabolites. The concentration of extracellular metabolites were generally near or below detection limits (Table S1). Nevertheless, the excretion of metabolites by actively growing cells has important implications for the environment, where compounds synthesized by chemoautotrophs could serve as substrates for heterotrophs or potentially facilitate synergistic interactions (Azam & Malfatti, 2007; Durham et al., 2015; Stokke et al., 2015).

The reductive tricarboxylic acid cycle (rTCA) is used for autotrophic carbon fixation in *S. denitrificans* and other chemolithoautotrophic *Campylobacteria* (Hügler & Sievert, 2011; Hügler et al., 2005; Takai et al., 2006). The intracellular detection of four out of the five standards that are part of the rTCA cycle (citric acid, malic acid, fumaric acid, and succinic acid) underlines its central importance in *S. denitrificans*. While fumaric acid was present only at low concentrations, other intermediates of the rTCA cycle, such as malic acid and succinic acid, were present at higher concentrations. Another intermediate of the rTCA cycle, α‐ketoglutaric acid, could only be detected extracellularly, suggesting a rapid turnover inside the cell to glutamic acid. These differences in concentration among the rTCA cycle metabolites could be explained by the various involved enzymes with different reaction rates and substrate affinities (Hügler et al., 2005). Citric acid, which is a central metabolite of the rTCA cycle, was detected intracellularly in some, but not all samples. Because the concentrations of this metabolite were generally below the lowest standard level, citric acid could not be reliably quantified and is thus not included in Figure 1. These low levels of citric acid could be the result of its fast conversion into oxaloacetate by the ATP citrate lyase, the key enzyme of the rTCA cycle (Hügler et al., 2005).

Amino acids were also among the most abundant internal metabolites. Nine out of the 13 targeted amino acids were quantified (not identified were arginine, cysteine, threonine, and serine). These include proline, leucine/isoleucine, phenylalanine, tryptophan, tyrosine, aspartic acid, glutamic acid, methionine, and glutamine (Figure 1). All these amino acids are likely to be involved in protein synthesis, although some may have additional functions as discussed below. Precursors for amino acids like D(‐)3‐phosphoglyceric acid, which is part of the methionine synthesis pathway, were also detected (Tong et al., 2014). In addition, primary metabolites of DNA and RNA synthesis, including thymidine, guanine, adenine, cytosine, and uracil were observed, in line with active growth of *S. denitrificans* in the chemostat.

### 3.2 Adaption to changes in salinity

**Figure 3:**
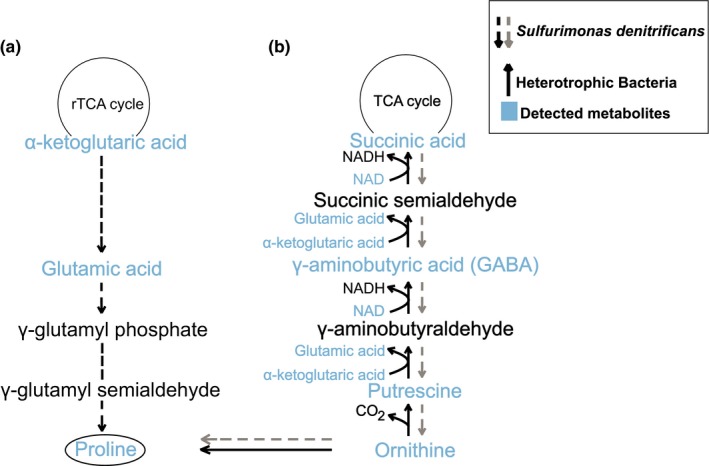
(a) Schematic diagram of the proposed synthesis pathway for proline in *Sulfurimonas denitrificans* (black dashed arrows; genes encoding for all involved enzymes are present in genome) and (b) the known pathway for succinic acid production from ornithine in heterotrophic bacteria (solid arrows) and the hypothetical pathway in *S. denitrificans* (gray dashed arrows; genes could not be confirmed in genome)

*S. denitrificans* was originally isolated from tidal mudflats (Timmer‐Ten Hoor, 1975), which are subject to periodic changes in salinity, requiring specific adaptations for osmoregulation. In line with this, we demonstrate here that *S. denitrificans* grows over a relatively wide range of ionic strengths from 180 to 520 mM (0–20 g/L NaCl). Furthermore, several metabolites occurring at high concentrations inside the cell also exhibited significant correlations with chloride concentration (Figure 2), suggesting that they may play a role in osmoregulation. Proline was among the metabolites with the highest internal concentration and the strongest correlation with increasing salinity (Figures 1 and 2). This indicates that proline could serve as an osmolyte in *S. denitrificans* similar to what has been observed in other bacteria (Brill, Hoffmann, Bleisteiner, & Bremer, 2011; Christian, 1955a,b; Fichman et al., 2014; Liang, Zhang, Natarajan, & Becker, 2013; Sleator & Hill, 2001). Glutamic acid (glutamate) is a well‐known precursor for proline synthesis (Fichman et al., 2014). All genes required for the biosynthesis of glutamic acid and further biosynthesis to proline are present in *S. denitrificans* (Sievert et al., 2008), and it also exhibited relatively high internal concentrations. Therefore, we suggest that the biosynthesis of proline proceeds from glutamic acid (Figure 3a). Unlike proline, however, glutamic acid concentration did not change with salinity. This is in contrast to what has been previously observed in other microorganisms that exhibit an increase in proline at the expense of glutamic acid with increasing sodium chloride concentrations (Saum & Müller, 2007).

In addition to glutamic acid, ornithine is another potential precursor to proline (Figure 3b). Putrescine, gamma aminobutyric acid, and glutamic acid were all detected in this study, suggesting that *S. denitrificans* may utilize multiple precursors for proline, which would facilitate a rapid metabolic response to osmotic stress. Although ornithine concentrations measured here were low, metabolites involved in ornithine synthesis were detected. Therefore, the synthesis of proline via ornithine via a reverse pathway that is known from heterotrophic bacteria cannot be excluded, although it is unlikely as no homologous genes could be detected in the genome of *S. denitrificans* (Feehily et al., 2014; Schneider, Kiupakis, & Reitzer, 1998) (Figure 3b). However, further work is required to assess the relative importance of the different pathways, for example, by measuring fluxes and determining expression of genes and proteins.

Another key intermediate of the rTCA cycle, α‐ketoglutaric acid, also appears to play a role in the osmoregulation of *S. denitrificans* by providing biosynthetic precursors for osmolytes (Fichman et al., 2014; Schneider et al., 1998). Three enzymes are known to be able to convert α‐ketoglutaric acid. The rapid conversion of α‐ketoglutaric acid to isocitrate by the isocitrate dehydrogenase, with the fastest specific activity in the rTCA cycle of *S. denitrificans* (Hügler et al., 2005), could explain why α‐ketoglutaric acid was not detected internally, though it was detected in some cases among the extracellular metabolites. The other two enzymes known to consume α‐ketoglutaric acid are putrescine transaminase and glutamic acid‐succinic semialdehyde transaminase, the latter of which converts α‐ketoglutaric acid into glutamic acid (Schneider et al., 1998). This enzyme could convert α‐ketoglutaric acid into a large internal pool of glutamic acid to enable the organism to react quickly to changes in the salt concentration by producing proline (Brill et al., 2011; Fichman et al., 2014; Liang et al., 2013). However, genes coding for either enzyme could not be identified in the genome of *S. denitrificans*.

**Figure 4:**
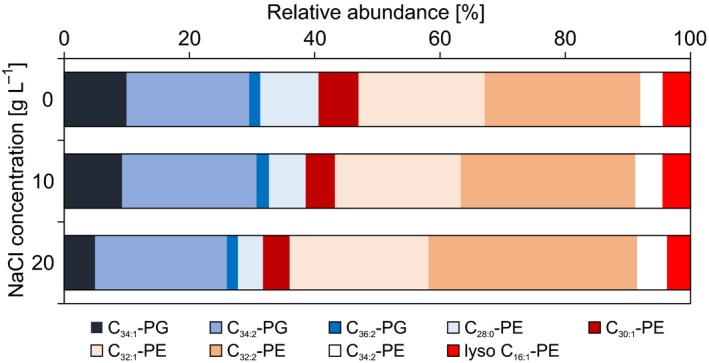
Relative abundance of dominant PE (phosphatidylethanolamine) and PG (phosphatidylglycerol) lipids at different NaCl concentrations (0, 10, and 20 g/L). Individual PG‐ and PE‐DAG lipids were identified by the total number of acyl carbons and double bonds (C_*n*:*m*_, where *n* indicates the number of carbon atoms and *m* the number of double bonds). The detailed molecular structure of fatty acids cannot be identified by positive ion HPLC‐MS alone

Another compound that showed a strong correlation with increasing salt concentration is 2,3‐dihydroxypropane‐1‐sulfonate (DHPS) (Figure 2). DHPS is a known degradation product of sulfoquinovose (Denger et al., 2014), which constitutes the headgroup of the sulfolipid sulfoquinovosyldiacylglycerol from the membrane of photosynthetic eukaryotes and bacteria and is also a component of the S‐layer of archaea (Denger et al., 2014; Meyer et al., 2011; Van Mooy et al., 2009). Heterotrophic bacteria are also able to degrade DHPS to pyruvate and/or acetyl‐CoA (Durham et al., 2015; Mayer et al., 2010). However, the analysis of the genome of *S. denitrificans* revealed no evidence for the presence of a pathway for the degradation or synthesis of sulfoquinovose, or for the ability to produce sulfolipids. This latter aspect was further tested by analyzing the lipidome of *S. denitrificans* using HPLC‐MS. Phosphatidylethanolamine (PE) and the phosphatidylglycerol (PG) were the only detected polar membrane lipids in *S. denitrificans* (Figure 4). Furthermore, in response to increasing salt concentration, *S. denitrificans* increases the level of unsaturation of the fatty acids, which is shown by a higher relative abundance of mono‐ and diunsaturated lipids and lower abundance of saturated lipids at higher salinities (Figure 4). This strategy is well‐known from other bacteria (Russell, 1989). However, in line with the genomic analysis, we were unable to identify any sulfolipids in *S. denitrificans*. Future work is needed to address the origin of DHPS in *S. denitrificans* and to assess its potential role in the adaptation to osmotic stress or any other presently unknown function.

Phenylalanine also showed a significant correlation with changing salt concentrations (Figure 2). However, at present there are no reports indicating that bacteria might produce phenylalanine for osmoregulation or in response to any other stressors. In previous studies of cyanobacteria, phenylalanine was found at consistently higher levels outside the cell (Fiore et al., 2015), but in this study extracellular phenylalanine concentrations were near detection limits. Intracellular tyrosine, which is formed from phenylalanine, also showed a positive, albeit weak, correlation with increasing salt concentration. Further studies are needed to investigate a novel function of phenylalanine and possibly tyrosine in mechanisms for osmoregulation in *S. denitrificans*.

## 4 CONCLUSIONS

A targeted metabolomics approach was used to investigate osmoregulation in the chemolithoautotrophic campylobacterium *Sulfurimonas denitrificans*. The quantified metabolites highlight the central importance of the rTCA cycle, not only for carbon fixation, but also as a hub for the precursors of other anabolic pathways, including the production of the osmolyte proline from α‐ketoglutaric acid (via glutamic acid). Proline was identified as the major osmolyte allowing *S. denitrificans* to adapt to osmotic stress. In addition, the intracellular concentration of DHPS was significantly correlated with increasing salt concentration, suggesting that DHPS may also be involved in osmoregulation. However, no genes could be identified in the genome of *S. denitrificans* that encode enzymes known to produce DHPS via sulfolipid degradation. Possibly, the excretion of metabolites by actively growing cells might facilitate growth by heterotrophs or synergistic interactions in the environment. Our metabolomics approach not only provides important baseline information to help inform future transcriptomic, proteomic, and metabolomic studies of *Campylobacteria*, but also underscores the utility of metabolomics for revealing new biochemical information and adaptations not readily apparent based on genome analysis alone.

## CONFLICT OF INTEREST

None declared.
