# Supplementary material for: Targeted metabolomics reveals proline as a major osmolyte in the chemolithoautotroph Sulfurimonas denitrificans
Source: Microbiologyopen. 2018 Feb 9;7(4):e00586. doi: 10.1002/mbo3.586 (PMC6079173; doi:10.1002/mbo3.586)
Supplement: Supplementary file 4 [file MBO3-7-e00586-s004.docx]

**Table S1** Extracellular metabolites detected in supernatants of replicate chemostats (C1, C2, and C3) at different sodium chloride concentrations (0, 10 and 20 g L^-1^). Concentrations are given in picogram per milliliter (pg mL^-1^). Blanks in the table indicate that the metabolite was not detected.

| Replicate  NaCl [g L^-1^] | C1  0 | C2  0 | C3  0 | C1  10 | C2  10 | C3  10 | C1  20 | C2  20 | C3  20 |
| --- | --- | --- | --- | --- | --- | --- | --- | --- | --- |
| Thymidine | 22,475 | 20,371 | 10,864 | 33,164 | 83,537 | 17,003 | 19,247 | 29,810 | 16,544 |
| Glucose 6-phosphate | 6,609 | 19,662 | 25,303 | 9 |  | 2,272 | 3,937 |  | 2,507 |
| Guanine |  | 594 | 7,871 | 1,420 | 2,091 | 684 | 3,081 |  | 1,710 |
| Gamma aminobutyric acid |  | 445 | 6,270 | 663 | 1,788 | 1,190 | 2,778 | 2 | 1,262 |
| Phenylalanine |  |  | 15,696 | 198 | 235 |  | 2,615 | 1,515 | 1,241 |
| Sarcosine | 1,152 | 226 | 642 | 1,791 | 3,660 | 537 | 1,879 | 551 | 404 |
| Tyrosine | 483 | 594 | 370 | 220 | 666 | 325 | 851 | 277 | 534 |
| 2,3-Dihydroxypropane 1-sulfonate | 983 | 1,270 | 1,466 | 420 | 158 | 1,439 | 753 |  | 302 |
| Cytosine | 263 | 291 | 2,149 | 1,765 | 6,079 | 891 | 649 |  | 347 |
| 5'-Adenosine monophosphate | 172 | 885 | 1,329 | 380 | 922 | 33 | 628 | 15 | 463 |
| D-ribose 5-phosphate | 530 | 777 |  | 92 |  | 904 | 499 |  | 181 |
| n-Acetyl glucosamine | 127 | 1,025 | 956 |  |  | 911 | 495 |  | 148 |
| Sodium taurocholate | 649 | 615 | 797 | 434 | 782 | 713 | 361 | 187 | 330 |
| Proline | 661 | 318 | 100 | 182 | 346 | 4,455 | 321 | 46 | 172 |
| 2,3-Dihydroxybenzoic acid | 303 | 284 | 359 |  |  | 317 | 164 | 83 |  |
| Methionine | 13 | 419 | 245 | 254 | 905 | 176 | 162 | 0.5 | 81 |
| Uracil |  |  |  |  | 51 |  | 124 |  |  |
| Adenine | 9 | 36 | 2,881 |  |  |  | 113 |  | 98 |
| Xanthine | 471 | 130 | 6,357 | 47 | 747 | 704 | 98 | 3 | 104 |
| s-Adenosyl methionine |  |  |  |  |  |  | 55 | 18 |  |
| Riboflavin | 49 | 50 | 63 | 41 | 33 | 20 | 42 | 26 | 18 |
| Caffeine | 33 | 6 |  | 8 |  | 2 | 39 | 17 | 16 |
| Desthiobiotin | 36 | 34 | 87 | 24 | 41 | 44 | 25 | 10 | 23 |
| Indole 3-acetic acid | 39 | 56 | 68 |  |  | 46 | 21 |  | 20 |
| Biotin | 2 | 5 | 60 | 5 |  | 15 | 16 | 13 | 17 |
| 5'(methylthio)adenosine |  |  | 1 | 0.02 | 0.1 |  | 1 | 1 | 0.1 |
| Citric acid |  | 1,600 |  | 990 |  |  |  |  | 726 |
| D(-)3-Phosphoglyceric acid | 502 |  |  | 453 | 673 |  |  |  |  |

**Table S1 continued**

| Replicate  NaCl [g L^-1^] | C1  0 | C2  0 | C3  0 | C1  10 | C2  10 | C3  10 | C1  20 | C2  20 | C3  20 |
| --- | --- | --- | --- | --- | --- | --- | --- | --- | --- |
| Leucine/ Isoleucine | 437 |  |  | 72 |  |  |  |  |  |
| Folic acid |  |  | 95 | 53 |  | 85 |  | 23 |  |
| Succinic acid | 13,232 | 3,236 | 28,826 | 37 |  | 13,107 |  | 67 |  |
| Ornithine | 29 |  |  | 18 | 46 |  |  | 14 |  |
| Malic acid |  |  |  | 5 |  |  |  |  |  |
| Pantothenic acid | 32 | 32 | 46 | 2 |  | 2 |  | 1 | 6 |
| n-Acetyl glutamic acid | 996 | 562 | 409 | 0.4 |  | 827 |  | 21 |  |
| Nicotinamide adenine dinucleotide |  |  |  |  |  |  |  |  | 455 |
| α-Ketoglutaric acid | 819 |  | 562 |  | 11 | 1,291 |  | 21 | 53 |
| 4-Hydroxybenzoic acid | 128 | 72 | 52 |  |  | 57 |  |  | 31 |
| Putrescine | 60 |  | 27 |  | 9 |  |  |  | 27 |
| Tetrahydrobiopterin |  |  |  |  |  |  |  |  | 1 |
| Glutathione |  |  |  |  | 298 | 273 |  |  |  |
| sn-Glycerol 3-phosphate | 277 | 36 |  |  | 58 | 27 |  |  |  |
| Thiamine monophosphate |  | 2 | 0.5 |  | 4 | 6 |  | 0.1 |  |
| Cyanocobalamin |  |  |  |  | 1 |  |  | 0.2 |  |
| Fosfomycin | 512 | 454 |  |  |  |  |  |  |  |
| Orotic acid | 25 | 227 | 98 |  |  |  |  | 19 |  |
| Fumaric acid | 361 | 216 |  |  |  | 89 |  | 29 |  |
| 6-Phosphogluconic acid |  | 71 |  |  |  | 311 |  |  |  |
| Tryptophan |  |  | 29 |  |  |  |  |  |  |
